# Ampelopsis Radix Protects Dopaminergic Neurons against 1-Methyl-4-phenylpyridinium/1-methyl-4-phenyl-1,2,3,6-tetrahydropyridine-Induced Toxicity in Parkinson's Disease Models *In Vitro* and *In Vivo*

**DOI:** 10.1155/2013/346438

**Published:** 2013-09-24

**Authors:** Hanbyeol Park, Jin Sup Shim, Hyo Geun Kim, Hyejung Lee, Myung Sook Oh

**Affiliations:** ^1^Department of Life and Nanopharmaceutical Science, Kyung Hee University, No. 1 Hoegi-dong, Dongdaemun-gu, Seoul 130-701, Republic of Korea; ^2^Department of Oriental Pharmaceutical Science, College of Pharmacy and Kyung Hee East-West Pharmaceutical Research Institute, Kyung Hee University, No. 1 Hoegi-dong, Dongdaemun-gu, Seoul 130-701, Republic of Korea; ^3^Studies of Translational Acupuncture Research (STAR), Acupuncture & Meridian Science Research Center (AMSRC), Kyung Hee University, No. 1 Hoegi-dong, Dongdaemun-gu, Seoul 130-701, Republic of Korea

## Abstract

Ampelopsis Radix, the root of *Ampelopsis japonica* (Thunb.) Makino (Vitaceae), is a herbal medicine which has been widely used in East Asia. The present study was done to explore whether the standardized extract of Ampelopsis Radix (AJW) protects dopaminergic neurons via antioxidant mechanisms in Parkinson's disease (PD) models. The effects of AJW on primary mesencephalic cultures stressed with 1-methyl-4-phenylpyridinium were investigated using tyrosine hydroxylase (TH) immunohistochemistry and reactive oxygen species measurement. The eliminative effects of AJW on the 2,2-diphenyl-1-picrylhydrazyl and 2,2′-azino-bis-(3-ethylbenzthiazoline-6-sulphonic acid) radicals were explored using colorimetric methods. The effects of AJW on the mice treated with 1-methyl-4-phenyl-1,2,3,6-tetrahydropyridine (MPTP) were determined by pole test as well as TH and 8-hydroxydeoxyguanosine immunohistochemistry. AJW protected dopaminergic neurons by inhibiting reactive oxygen species generation *in vitro*. Moreover, AJW showed potent radical scavenging activities *in vitro*. In the mouse PD model, AJW protected the dopaminergic neurons in the brain, leading to motor improvements. AJW inhibited the MPTP-evoked accumulation of 8-hydroxydeoxyguanosine in the brain. These data suggest that AJW has neuroprotective effects with antioxidant mechanisms in PD models.

## 1. Introduction

Oxidative stress occurs as a result of imbalance of the free radical generation and antioxidant defense system [1]. Even under natural physiological states, however, the oxygen consumption by aerobes, which is necessary for their immediate survival, leads to the potentially detrimental reactive oxygen metabolites generation [2]. The brain is deemed to be vulnerable to oxidative damage because of its high metabolic rate and relatively low cellular regeneration ability compared with other organs [3]. In addition, dopaminergic neurons in the substantia nigra pars compacta (SNc) are thought to be more prone to oxidative stress because the self-metabolism of dopamine generates reactive oxygen species (ROS), including various peroxides and radicals [4]. Therefore, oxidative stress might contribute to the pathogenesis of Parkinson's disease (PD), a common degenerative brain disease, which is featured by selective dopaminergic neurodegeneration from the SNc to the striatum and by clinical symptoms of bradykinesia, resting tremor, and rigidity [5]. Supporting this oxidative stress hypothesis, considerable levels of oxidative damaged macromolecules such as proteins, lipids, and DNA have been reported in PD brains [6].

As a very common neurotoxin for PD models, 1-methyl-4-phenyl-1,2,3,6-tetrahydropyridine (MPTP) causes clinical and chemical alterations in humans and rodents similar to those that occur in PD [7, 8]. After administration, MPTP rapidly passes through the brain and is converted into its active form, 1-methyl-4-phenylpyridinium (MPP^+^), by an oxidizing enzyme in astrocytes [9]. MPP^+^ is transferred to dopaminergic neurons through the dopamine transporter, where it inhibits the respiratory chain by disrupting mitochondrial complex I, and leads to oxidative stress via mitochondria-dependent or -independent pathways [10]. The oxidative stress generated by MPP^+^ subsequently results in the peroxidation of macromolecules, ultimately resulting in cell death [10].

Ampelopsis Radix, the root of *Ampelopsis japonica* (Thunb.) Makino, belongs to the grape family (Vitaceae) and has been widely used as a traditional medicine to treat pain and fever in East Asia [11]. It contains well-known bioactive components, including resveratrol, schizandriside, catechins, and other polyphenol compounds [11, 12]. Researches on (+)-catechin and (−)-epicatechin demonstrated that they have the strong free radical eliminating activities and protective effects against 6-hydroxydopamine (6-OHDA) neurotoxicities [13, 14]. (+)-Catechin further exerted neuroprotective effects via antioxidant mechanisms against 6-OHDA- or MPP^+^-induced toxicities in various cellular systems [15]. In addition, resveratrol protects neurons in an injured-spinal-cord rat model with its antioxidant properties, which increase superoxide dismutase activity and decrease malondialdehyde levels [16]. Nevertheless, few studies have examined the pharmacological properties of Ampelopsis Radix, and particularly the possible protective effects in PD models have not been demonstrated. Therefore, we explored the effects of an aqueous extract of Ampelopsis Radix on mesencephalic dopaminergic cells stressed with MPP^+^ and C57BL/6 mice stressed with MPTP. We also examined the possible antioxidant mechanisms of this protection.

## 2. Materials and Methods

### 2.1. Materials

Minimal essential medium was purchased from Gibco (Carlsbad, CA, USA). Fetal bovine serum (FBS) was purchased from Hyclone Lab Inc. (Logan, UT, USA). (+)-Catechin, (−)-epicatechin, 2,2-diphenyl-1-picrylhydrazyl (DPPH), 2,2′-azino-bis-(3-ethylbenzthiazoline-6-sulphonic acid) (ABTS), potassium persulfate, poly-L-lysine (PLL), glucose, glutamine, MPTP, MPP^+^, 2,7-dichlorodihydrofluorescein diacetate (H_2_DCF-DA), phosphate-buffered saline (PBS), paraformaldehyde (PFA), 3,3-diaminobenzidine (DAB), sucrose, and bovine serum albumin (BSA) were purchased from Sigma-Aldrich (St. Louis, MO, USA). Rabbit anti-tyrosine hydroxylase (TH) antibody was purchased from Chemicon International Inc. (Temecula, CA, USA). Mouse anti-8-hydroxydeoxyguanosine (8-OHdG) antibody was purchased from JaICA (Shizuoka, Japan). Biotinylated anti-rabbit and anti-mouse antibodies, normal goat serum, and avidin-biotin-peroxidase complex (ABC) standard kit were purchased from Vector Lab (Burlingame, CA, USA). Zoletil was purchased from Virbac (Carros, France).

### 2.2. Preparation of Extract and Standardization

A dried root of *A. japonica* was obtained from Jung Do Hrbal Drug Co. (Seoul, Republic of Korea). The voucher specimen (KHUOPS-MH023) was deposited in the herbarium of the College of Pharmacy, Kyung Hee University (Seoul, Republic of Korea). It was extracted with distilled water at 100°C for 2 h. The powder (AJW) was made from the extracts by filtering and lyophilization. The yield of AJW was 16.10% and it was kept at 4°C. AJW was standardized with (+)-catechin and (−)-epicatechin known as constituents in *A. japonica *[11], using reverse-phase high-performance liquid chromatography system consisting of a Waters (Miliford, MA, USA) model 515 pump, a 717 autosampler, and a 996 photodiode array detector. Separation was conducted using Atlantis C18 column (150 × 4.6 mm, 3 *μ*m; Waters, Miliford, MA, USA) at 25°C. Mobile phases included aqueous solution of 0.2% acetonitrile (eluent M1) and methanol (eluent M2). The gradient elution was carried in the way that the eluent M2 was linearly increased from 0 to 50% for 0 to 24 min and from 50 to 100% for 24 to 40 min at a flow rate of 1.2 mL/min. The injection volume was 10 *μ*L, and the detector wavelength was set at 280 nm. AJW and reference to the calibration curve obtained with (+)-catechin and (−)-epicatechin were analyzed in triplicates. (+)-Catechin and (−)-epicatechin were found in AJW at a mean level of 1.81 ± 0.16 mg/g and 1.40 ± 0.04 mg/g, respectively.

### 2.3. Primary Cultures of Rat Mesencephalic Cells

Mesencephalic cell cultures were derived from 14-day embryos of Sprague-Dawley rat (Daehan Biolink Co., Eumseong, Republic of Korea). Mesencephalons were dissected, collected, dissociated, and seeded in PLL precoated 24-well plates at a density of 1.0 × 10^5^ cells/well. Cultures were maintained in a humidified incubator of 5% CO_2_ at 37°C in minimal essential medium with 6 g/L glucose, 2 mM glutamine, and 10% FBS. On the 6th day *in vitro*, cells were treated with AJW for 1 h and stressed with 12 *μ*M MPP^+^ for a further 23 h. An equal volume of vehicles was given to the control and toxin groups. Then, cells were fixed with 4% PFA at room temperature for 30 min. The cells were stored in PBS at 4°C for immunohistochemistry.

### 2.4. Determination of Intracellular ROS Level

Intracellular ROS was measured using a fluorescent probe, H_2_DCF-DA. Intracellular H_2_O_2_ or low-molecular weight peroxides oxidize H_2_DCF-DA to the highly fluorescent compound dichlorofluorescein (DCF). Cells were seeded in PLL precoated 24-well plates at a density of 1.0 × 10^5^ cells/well, and on the 6th day *in vitro*, cells were treated with AJW for 1 h and stressed with 12 *μ*M MPP^+^ for a further 23 h. An equal volume of vehicles was given to the control and toxin groups. Cells were incubated with 20 *μ*M H_2_DCF-DA at 37°C for 30 min, the cells on cover slips were mounted on gelatin-coated glass slides; they were photographed with a fluorescence microscope (BX51T-32F01; Olympus Co., Tokyo, Japan). The fluorescence intensity of DCF was measured by a fluorescence microscope, and was expressed as a percentage of the value in the vehicle-treated control group.

### 2.5. Measurement of DPPH Radical Scavenging Activity

AJW at various concentrations of 1–1000 *μ*g/mL was mixed with 0.2 mM DPPH ethanol solution (1 : 1). After incubation at room temperature in the dark for 30 min; the absorbance of the mixture was determined at 517 nm using spectrophotometer (VersaMax microplate reader; Molecular Device, Sunnyvale, CA, USA). Also, the activity of AJW was expressed as half maximal inhibiting concentration (IC_50_) which is defined as the concentration of AJW required to scavenge 50% of DPPH radicals. IC_50_ values were estimated by a nonlinear regression using the GraphPad Prism software (GraphPad Software Inc., San Diego, CA, USA).

### 2.6. Measurement of ABTS Radical Cation Scavenging Activity

ABTS solution of 7.4 mM was added to 2.6 mM potassium persulfate for 1 day before starting the experiment in the dark. AJW at various concentrations of 1–1000 *μ*g/mL was mixed with 7.4 mM ABTS solution with 2.6 mM potassium persulfate. After incubation at room temperature for 5 min, the absorbance of the mixture was determined at 732 nm using spectrophotometer. Also, the activity of AJW was expressed as IC_50_ which is defined as the concentration of AJW required to scavenge 50% of ABTS radical cations. IC_50_ values were estimated by a nonlinear regression using the GraphPad Prism software.

### 2.7. Animals and Treatment

Animal maintenance and treatment were carried out in accordance with the Principles of Laboratory Animal Care (NIH publication number 85-23, revised 1985) and the Animal Care and Use guidelines of Kyung Hee University, Seoul, Republic of Korea. Male C57BL/6 mice (8 weeks old) were purchased from Daehan Biolink. Animals were assigned to four groups: (1) Group 1 (vehicle-treated group), (2) Group 2 (MPTP-only treated group), (3) Group 3 (MPTP + AJW 10 mg/kg/day treated group), and (4) Group 4 (MPTP + AJW 100 mg/kg/day treated group). The mice were housed at an ambient temperature of 23 ± 1°C and a relative humidity of 60 ± 10% under a 12-h light/dark cycle and were allowed free access to water and food. AJW dissolved in saline was administered orally at 10 or 100 mg/kg/day for 7 days. MPTP (MPTP base form; 20 mg/kg) in saline was injected intraperitoneally four times at 2 h intervals on the last day of AJW treatment.

### 2.8. Behavioral Test and Brain Tissue Preparation

We performed the pole test, on the seventh day after the last MPTP injection. The mice were placed head upward near the top of a vertical rough-surfaced pole (diameter 8 mm, height 55 cm). The time taken for the mice to turn completely downward (time to turn, T-turn) and the time taken to reach the floor (locomotion activity time, T-LA) were recorded, with a cut-off limit of 30 sec. After the pole test, the mice were anesthetized with 50 mg/kg Zoletil (intramuscularly) and were rapidly perfused transcardially with PBS, followed by 4% PFA in 0.1 M phosphate buffer (PB). Then, brains were rapidly taken out, postfixed in 4% PFA, and processed for cryoprotection in 30% sucrose at 4°C. Frozen brains were cut into 30 *μ*m coronal sections using a cryostat microtome (CM3000; Leica, Wetzlar, Germany). Then, the tissues were stored in storing solution containing glycerin, ethylene glycol, and PB at 4°C for immunohistochemistry.

### 2.9. Immunohistochemistry

Fixed mesencephalic cells on cover slips and free-floating brain sections were rinsed in PBS at room temperature before immunostaining. They were pretreated with 1% H_2_O_2_ in PBS for 15 min. For dopaminergic neuron detection, they were incubated with a rabbit anti-TH antibody (1 : 2000 dilution) overnight at 4°C. For detection of oxidative DNA damage, the treated brain sections were heated in citric acid (0.1 M, 90°C) for 5 min for antigen retrieval, incubated in blocking solution (10% goat serum + 1% BSA in PBS) for 1 h, and incubated with mouse anti-8-OHdG antibody (1 : 50 dilution in blocking solution) overnight at 4°C. They were then incubated with a biotinylated anti-mouse and anti-rabbit IgG, respectively, for 1 h followed by incubation in ABC solution for 1 h at room temperature. The activity was visualized with DAB for 3 min. After every incubation step, the cells and tissues were washed three times with PBS. Finally, the mesencephalic cells on cover slips were mounted on gelatin-coated glass slides and air-dried. The free-floating brain tissues were mounted on gelatin-coated slides, dehydrated, cleared with xylene, and cover-slipped using histomount medium. All of them were photographed with an optical light microscope (BX51T-32F01; Olympus Co.). For quantification of the effect of AJW in the mesencephalic dopaminergic cells, TH-positive cells were counted on at least four cover slips from independent experiments for each condition. Quantification of the effect of AJW in brain tissues was performed by counting the TH-positive cell numbers and the 8-OHdG-positive cell numbers in the SNc at ×100 magnification under a microscope and by measuring the optical density of TH-positive fibers in the striatum at ×40 magnification using an ImageJ software (Bethesda, MD, USA). Data were expressed as a percentage of the value in the vehicle-treated control group.

### 2.10. Statistical Analysis

The data were expressed as mean ± standard error of the mean (SEM). Statistical significance was determined by one-way analysis of variance, followed by Tukey's multiple comparison test, using GraphPad Prism software. In all analyses, *P* < 0.05 was deemed to indicate statistical significance.

## 3. Results

### 3.1. Protective Effects of AJW against MPP^+^-Induced Toxicity in Primary Dopaminergic Neurons

**Figure 1:**
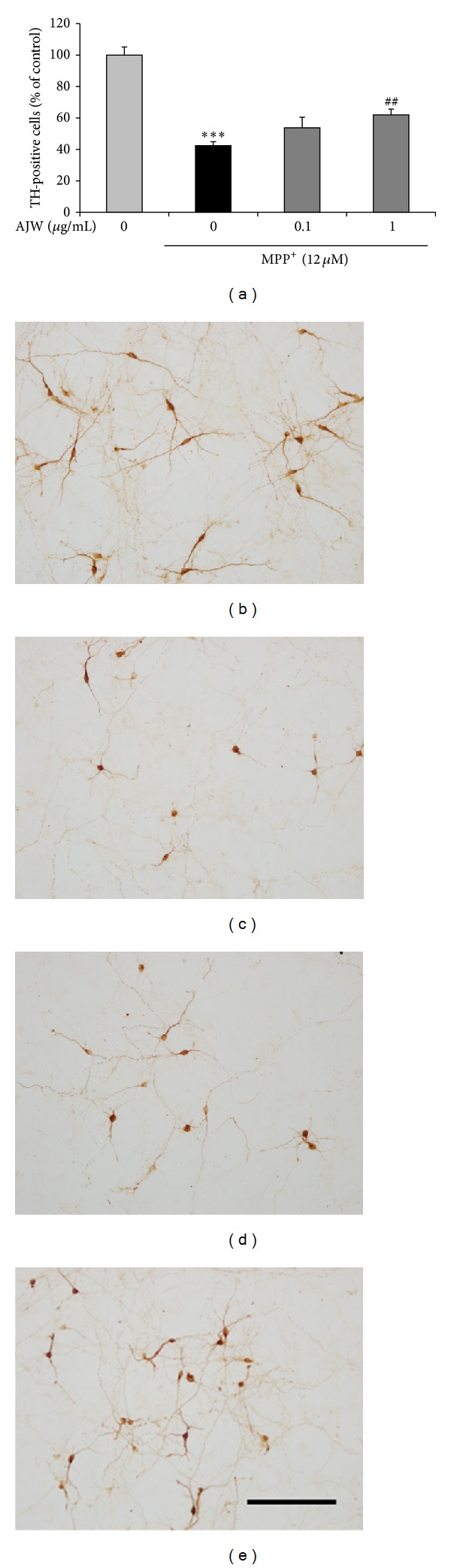
Protective effects of AJW against MPP^+^-induced toxicity in primary dopaminergic neurons. Cells were treated with AJW at 0.1, 1 *μ*g/mL, or vehicle for 1 h before exposure to 12 *μ*M MPP^+^ for 23 h. The numbers of TH-positive neurons were counted (a), and the representative images are shown ((b)–(e)). Control group (b), MPP^+^-only treated group (c), MPP^+^ + AJW 0.1 *μ*g/mL treated group (d), and MPP^+^ + AJW 1 *μ*g/mL treated group (e). Scale bar = 100 *μ*m. Values are indicated as the mean ± SEM. ***P < 0.001 compared with the control group, ^##^ *P* < 0.01 compared with the MPP^+^-only treated group.

To evaluate the protective effects of AJW against MPP^+^ toxicity in primary dopaminergic neurons, we counted TH-positive cell numbers. Exposure to 12 *μ*M MPP^+^ exhibited remarkably reduced numbers of TH-positive cells as 42.50 ± 2.45% relative to the control group and the treated cells had shrunken bodies and shortened neurites. Treatment of AJW at 1 *μ*g/mL protected dopaminergic cells, showing increased survival rate by 61.96 ± 3.70% relative to the control group and better neuronal morphology of  TH-positive cells (Figure 1).

### 3.2. Inhibitory Effects of AJW on ROS Production by MPP^+^ in Primary Mesencephalic Cells

**Figure 2:**
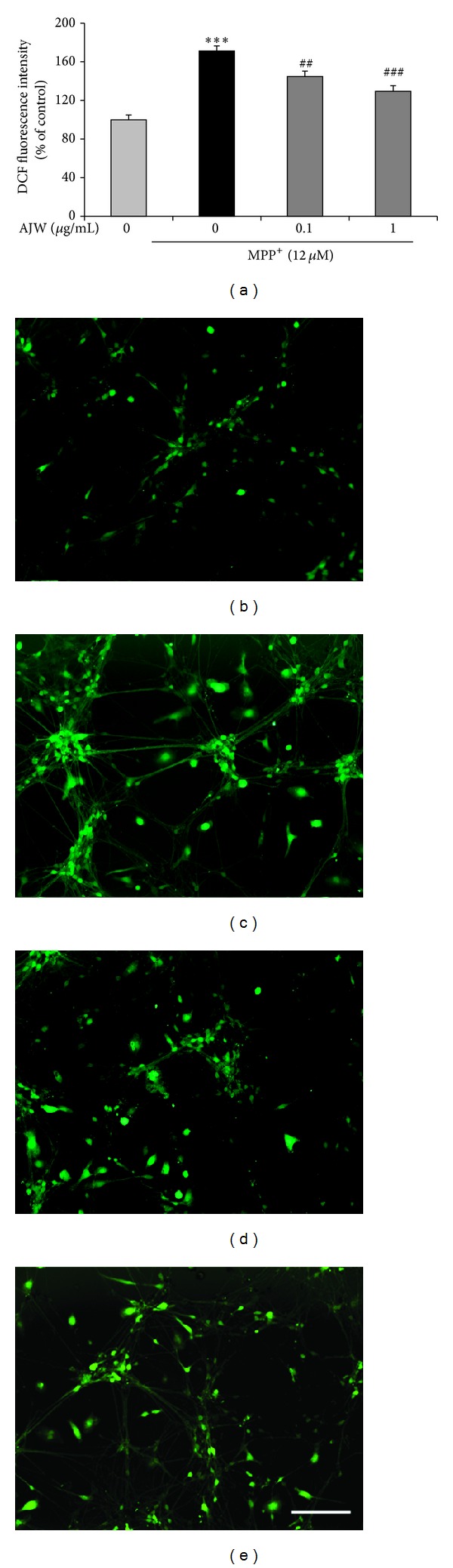
Inhibitory effects of AJW on ROS generation by MPP^+^ in primary mesencephalic cells. Cells were treated with AJW at 0.1, 1 *μ*g/mL, or vehicle for 1 h before exposure to 12 *μ*M MPP^+^ for 23 h. The fluorescence intensity of DCF was measured (a), and the representative images are shown ((b)–(e)). Control group (b), MPP^+^-only treated group (c), MPP^+^ + AJW 0.1 *μ*g/mL treated group (d), and MPP^+^ + AJW 1 *μ*g/mL treated group (e). Scale bar = 100 *μ*m. Values are indicated as the mean ± SEM. ***P < 0.001 compared with the control group, ^##^ *P* < 0.01, and ^###^ *P* < 0.001 compared with the MPP^+^-only treated group.

To evaluate the inhibitory effects of AJW on ROS generation by MPP^+^ in primary mesencephalic cells, we used H_2_DCF-DA. Exposure to 12 *μ*M MPP^+^ exhibited significantly increased intensity of DCF by 171.15 ± 5.37% relative to the control group, while the treatment with AJW at 0.1 and 1 *μ*g/mL showed markedly decreased intensity by 144.87 ± 5.51 and 129.51 ± 5.79% relative to the control group, respectively (Figure 2).

### 3.3. Radical Scavenging Activities of AJW

**Figure 3:**
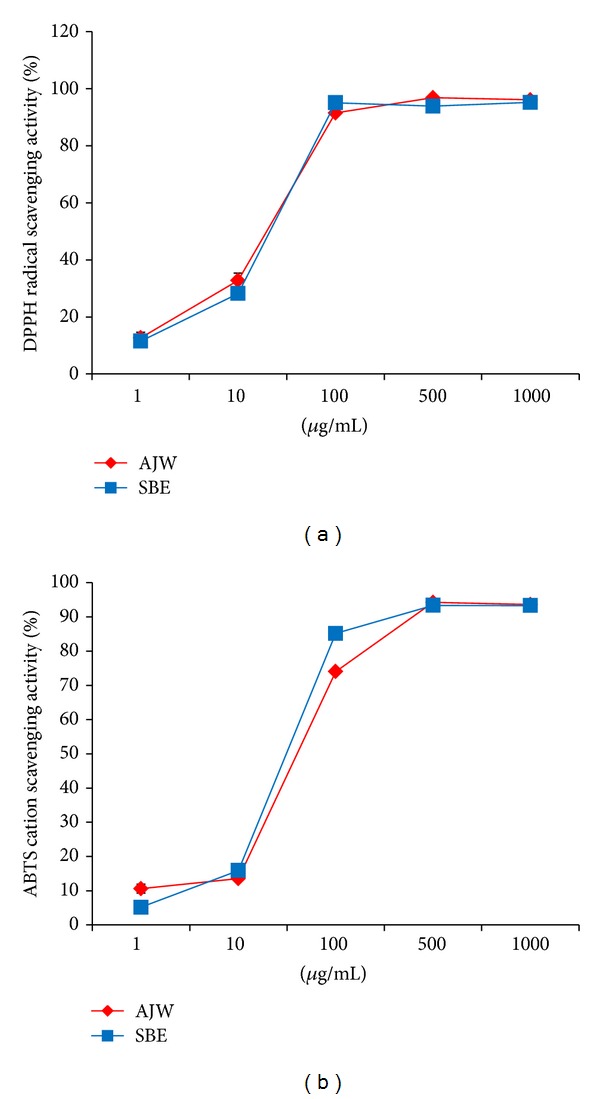
Radical scavenging activities of AJW. AJW and SBE, a positive control, showed DPPH free radical scavenging activity (a) and ABTS radical cation scavenging activity (b) in a dose-dependent manner at concentrations of 1–1000 *μ*g/mL. SBE = water extract of Scutellariae Radix. Values are indicated as the mean ± SEM.

To examine the antioxidant capacities of AJW, we performed DPPH free radical and ABTS radical cation scavenging activity assays. In both assays, AJW exhibited dose-dependently increased activities similar to the water extract of Scutellariae Radix (SBE), used as a positive control. AJW at 1000 *μ*g/mL exhibited 96.12 ± 0.52% and 93.64 ± 0.04% of scavenging activities in DPPH (Figure 3(a)) and ABTS (Figure 3(b)) assays, respectively. DPPH IC_50_s for AJW and SBE were 36.12 ± 2.79 and 39.28 ± 1.02 *μ*g/mL, whereas ABTS IC_50_s for AJW and SBE were 64.26 ± 0.06 and 54.30 ± 0.33 *μ*g/mL, respectively.

### 3.4. Inhibitory Effects of AJW on Behavioral Impairment by MPTP in Mice

**Figure 4:**
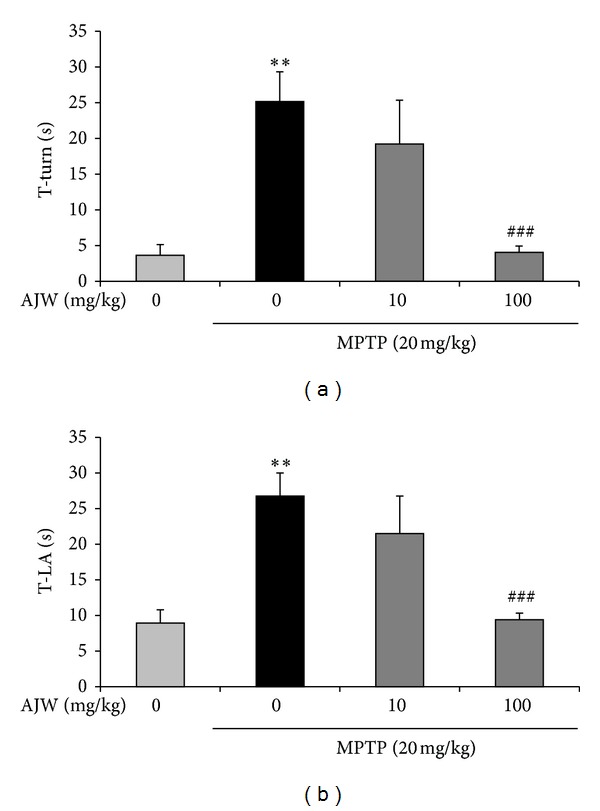
Inhibitory effects of AJW on behavioral impairment by MPTP in mice. Saline or AJW at 10 or 100 mg/kg/day was administered orally for 7 days, and MPTP 20 mg/kg was injected intraperitoneally four times at 2 h intervals on the last day of AJW treatment. Seven days after the last MPTP injection, we conducted the pole test. The time taken for the mice to turn completely downward ((a); T-turn) and the time taken to reach the floor ((b); T-LA) were recorded. Values are indicated as the mean ± SEM. **P < 0.01 compared with the control group, ^###^ *P* < 0.001 compared with the MPTP-only treated group.

To confirm the effect of AJW on dopaminergic neurons in an *in vivo* PD model, we treated mice with AJW and/or MPTP and carried out the pole test. As a result, T-turn and T-LA of MPTP-only treated mice were markedly prolonged as 25.15 ± 4.17 sec and 26.74 ± 3.26 sec, compared with the control group (T-turn: 3.64 ± 1.49 sec; T-LA: 8.91 ± 1.88 sec) (Figure 4). However, the times of AJW 10 mg/kg/day treated group were shortened as 19.20 ± 6.14 sec and 21.49 ± 5.28 sec, respectively, and the AJW 100 mg/kg/day treated group showed more shortened T-turn as 4.06 ± 0.87 sec and T-LA as 9.39 ± 0.94 sec (Figure 4).

### 3.5. Inhibitory Effects of AJW on Dopaminergic Neuronal Loss by MPTP in Mice

**Figure 5:**
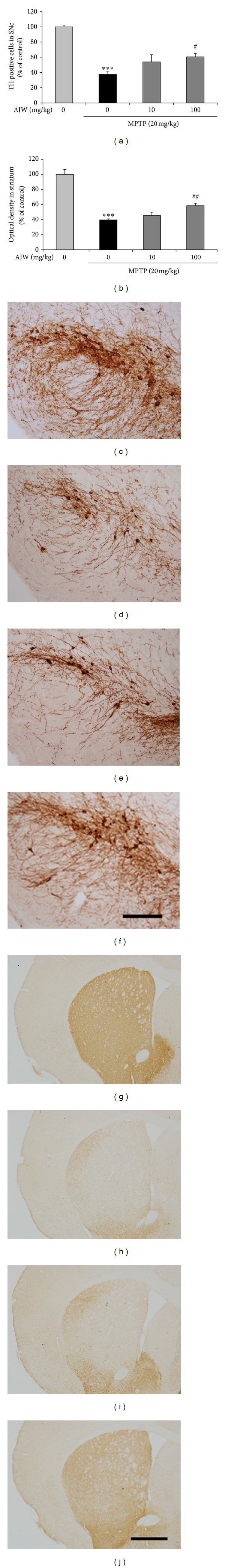
Protective effects of AJW on dopaminergic neuronal loss by MPTP in mice. Saline or AJW at 10 or 100 mg/kg/day was administered orally for 7 days, and MPTP 20 mg/kg was injected intraperitoneally four times at 2 h intervals on the last day of AJW treatment. After the behavioral test, dopaminergic neurons were determined using TH-immunohistochemistry. The numbers of TH-positive neurons in the SNc were counted (a) and the optical density of TH positive fibers in the striatum was measured (b). Representative images are shown of the SNc ((c)–(f)) and the striatum ((g)–(j)) of each group. Control group ((c) and (g)), MPTP-only treated group ((d) and (h)), MPTP + AJW 10 mg/kg/day treated group ((e) and (i)), and MPTP + AJW 100 mg/kg/day treated group ((f) and (j)). Scale bar = 100 *μ*m ((c)–(f)) and 400 *μ*m ((g)–(j)). Values are indicated as the mean ± SEM. ***P < 0.001 compared with the control group, ^###^ *P* < 0.001 compared with the MPTP-only treated group.

In the mouse brain, MPTP induced severe dopaminergic cell death in the SNc and the striatum. In the MPTP-only treated mice, the number of TH-positive cells in the SNc and the optical density in the striatum were decreased by 37.46 ± 3.46 and 39.53 ± 1.38% relative to the control group, respectively (Figure 5). However, these values are significantly increased by AJW 10 mg/kg/day treatment as 53.83 ± 9.54 and 45.34 ± 4.41% relative to the control group (Figure 5). Also, the values of AJW 100 mg/kg/day treated group more largely increased as 60.48 ± 4.68 and 58.48 ± 3.09% relative to the control group.

### 3.6. Inhibitory Effects of AJW on 8-OHdG Accumulation by MPTP in Mice

**Figure 6:**
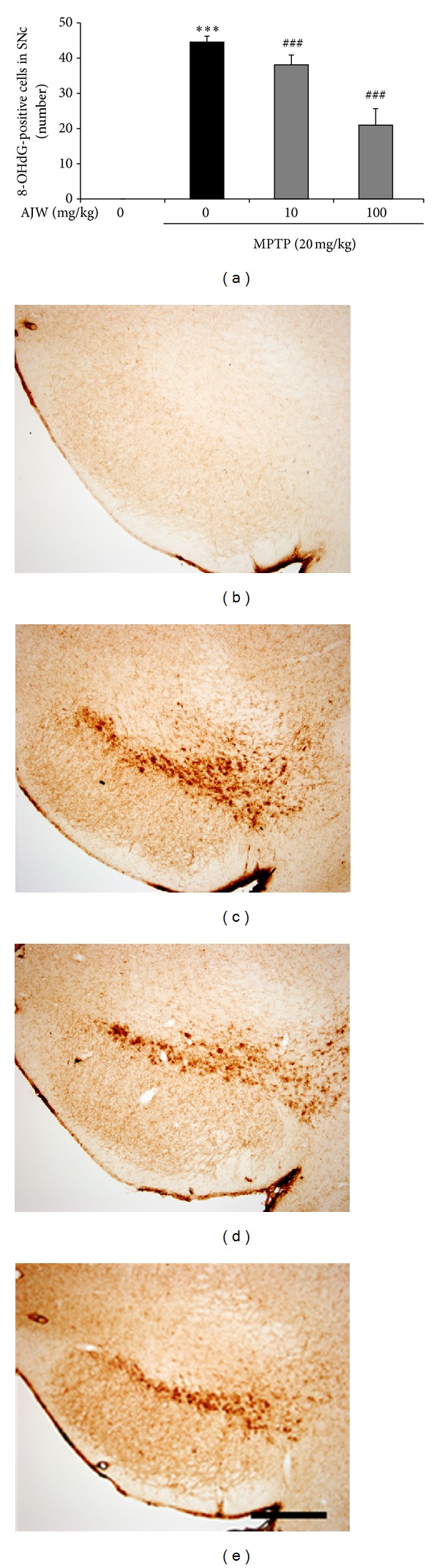
Inhibitory effects of AJW on 8-OHdG accumulation by MPTP in mice. Saline or AJW at 10 or 100 mg/kg/day were administered orally for 7 days, and MPTP 20 mg/kg was injected intraperitoneally four times at 2-h intervals on the last day of AJW treatment. After the behavioral tests, oxidative DNA damage in the cells of the SNc was detected using 8-OHdG-immunohistochemistry. The numbers of 8-OHdG-positive cells in the SNc were counted (a), and the representative images are shown ((b)–(e)). Control group (b), MPTP-only treated group (c), MPTP + AJW 10 mg/kg/day treated group (d), and MPTP + AJW 100 mg/kg/day treated group (e). Scale bar = 200 *μ*m. Values are indicated as the mean ± SEM. ***P < 0.001 compared with the control group, ^###^ *P* < 0.001 compared with the MPTP-only treated group.

To evaluate the inhibitory effects of AJW on 8-OHdG accumulation by MPTP in mice, we used anti-8-OHdG antibody. In the SNc of MPTP-only treated mice, large accumulation of 8-OHdG occurred, whereas in the control group, there is nothing shown (Figure 6). This accumulation is effectively prevented by treatment with AJW at 10 and 100 mg/kg/day as 85.48 ± 6.31 and 35.26 ± 13.92% of the value in the MPTP-only treated group (Figure 6).

## 4. Discussion

In this study, we demonstrated that AJW protects dopaminergic neurons against MPP^+^ toxicities in a primary culture system and against MPTP toxicities in mice through antioxidant mechanisms.

First, to investigate whether AJW protects dopaminergic neurons from MPP^+^ toxicity, we performed an immunohistochemistry of TH, the crucial enzyme in dopamine biosynthesis [17], in primary cultured mesencephalic cells. MPP^+^, the active metabolite of MPTP, is known to result in selective dopaminergic neuronal degeneration via inhibiting mitochondrial complex I and increasing calcium permeability of the mitochondrial membrane generating ROS in cellular PD models [18]. In this study, AJW remarkably protected dopaminergic neurons against MPP^+^ toxicity, leading to an increase in the TH-positive cell numbers and preservation of TH-positive cell morphologies.

Next, to explore the protective mechanisms of AJW from MPP^+^-induced neurotoxicity, we measured intracellular ROS level in primary cultured mesencephalic cells treated with MPP^+^ and/or AJW. ROS, generated mostly during the incomplete metabolic reduction of oxygen to water in aerobes, include superoxide, nitric oxide, hydroxyl radical, H_2_O_2_, and peroxynitrite [3]. All of these species are redox-active and can interact with nearby cellular components, resulting in wide-ranging damage [3]. Moreover, as one of the toxic events induced by MPP^+^, the overproduction of ROS readily damages dopaminergic neurons, which are especially vulnerable to oxidative stress [1, 19, 20]. In this study, AJW remarkably inhibited the intracellular ROS production triggered by MPP^+^ in primary mesencephalic cells. In addition, AJW showed potent radical scavenging activity comparable to SBE, a positive control that has strong antioxidant and neuroprotective effects [21], in DPPH and ABTS assays, suggesting that AJW scavenges the free radicals overproduced by MPP^+^.

Next, to evaluate the effects of AJW *in vivo*, we performed behavioral tests and brain tissue stereology after treating C57BL/6 mice with MPTP and/or AJW. The MPTP-treated mouse has been widely used as an *in vivo* PD model because MPTP induces PD-like motor deficits such as bradykinesia and rigidity and selective dopaminergic neuronal loss in the SNc [22]. In the pole test, which measures bradykinesia [23], AJW-treated mice showed significant improvement in the T-turn and T-LA to the level of the control group, compared with the MPTP-only treated group. We confirmed these effects of AJW in TH immunohistochemistry, showing that the AJW treatment protects both dopaminergic neurons in the SNc and its fibers in the striatum compared with the MPTP-only treated group. During MPTP-induced neurotoxicities, oxidative stress increases markedly and leads to the peroxidation of macromolecules [10]. Therefore, we examined the inhibitory effects of AJW on 8-OHdG accumulation in the SNc. 8-OHdG is the major DNA oxidative adduct, and it increases in the SNc and in the urine of PD patients [5]. Moreover, 8-OHdG levels in urine have been suggested to be a potential biomarker for investigating the progress of PD [5]. In our study, the MPTP-only treated mice showed significantly increased 8-OHdG accumulation in the SNc, whereas the control group showed nothing in the same region. However, AJW treatment prevented this accumulation, suggesting that AJW has inhibitory effects on the oxidative stress induced by MPTP. From these results, we confirmed that AJW protects dopaminergic neurons against MPTP-induced neurotoxicities through antioxidant mechanisms in PD models both *in vitro* and *in vivo*.

Most neurodegenerative diseases are heterogeneous and genetically complex. In PD, more than 90% of the cases are not linked to a single mutation [24]. Due to this multiplicity in the pathological processes of neurodegenerative diseases, materials that target more than one pathophysiological event in cell death cascades have been investigated [25]. And many drugs with single target often fail to show the expected effect in further studies [24]. However, because herbal medicines contain multiple compounds, they potentially could have multiple functions and produce stronger effects than a single compound via the synergism of their constituents [26]. Therefore, candidate herbal medicines have been evaluated recently [27]. For example, *Cassia obtusifolia* L. seed extract showed protective effects in experimental models of memory impairment, hippocampal cell death, ischemia, and PD [22]. Similarly, the root extract of *Polygala tenuifolia* Willd was reported to confer neuroprotection in various experimental models, such as memory impairment, cerebral ischemia, chronic stress, anxiety, and PD [17]. In addition, *Scutellaria baicalensis* Georgi root extract, another herbal medicine, protected neurons against ischemia, PD, Alzheimer's disease, and spinal cord injury in various experimental models [22]. Chemical profile of Ampelopsis Radix is rarely known, but Kim discovered that it contains resveratrol, (+)-catechin, (−)-epicatechin, schizandriside, (+)-gallocatechin, and (−)-epicatechin gallate, showing that (+)-catechin is the highest constituent in Ampelopsis Radix [11] and the content is similar to our result. Although the content of (+)-catechin in AJW, 0.18%, is not high, it could be enough to show activities in that (+)-catechin has been demonstrated to have strong neuroprotective activities in MPTP-treated mice even at 0.3 mg/kg [28] and to have good bioavailability [29]. In addition to (+)-catechin, (−)-epicatechin and resveratrol, which are known to be neuroprotective in various PD models [13, 15, 30], have been demonstrated to cross blood-brain barrier [31, 32]. Furthermore, the mixture of (+)-catechin and (−)-epicatechin was reported to have synergistic neuroprotective effects against amyloid *β* toxicities *in vitro* [33]. Taken together, the beneficial effects of AJW might be due to (+)-catechin mainly, and also other unknown compounds in AJW might synergistically contribute to the effects. Furthermore, those compounds could make AJW have potential to be applied to various neurodegenerative disease models, as the other herbal medicines mentioned above do. Nevertheless, the possible multimodal neuroprotective effects of AJW and the active compounds in AJW need to be further studied.

## 5. Conclusions

AJW protected dopaminergic cells from MPP^+^ toxicities in primary mesencephalic cultures and against MPTP toxicities in C57BL/6 mice. The neuroprotective effects of AJW are due to the antioxidant activities including inhibition of ROS, free radicals, and 8-OHdGs. These results indicated that AJW is a potential candidate as a neuroprotective agent in PD, although the major active ingredients in AJW and the possible protective effects of AJW in other PD models need to be further studied.
